# Advances toward the Elucidation of Hypertonic Saline Effects on *Pseudomonas aeruginosa* from Cystic Fibrosis Patients

**DOI:** 10.1371/journal.pone.0090164

**Published:** 2014-02-28

**Authors:** Anne-Laure Michon, Estelle Jumas-Bilak, Raphaël Chiron, Brigitte Lamy, Hélène Marchandin

**Affiliations:** 1 UMR 5119 ECOSYM, Equipe Pathogènes et Environnements, U.F.R. des Sciences Pharmaceutiques et Biologiques, Université Montpellier 1, Montpellier, France; 2 Laboratoire de Bactériologie, Hôpital Arnaud de Villeneuve, Centre Hospitalier Régional Universitaire de Montpellier, Montpellier, France; 3 Laboratoire d’Hygiène hospitalière, Centre Hospitalier Régional Universitaire de Montpellier, Montpellier, France; 4 Centre de Ressources et de Compétences pour la Mucoviscidose, Hôpital Arnaud de Villeneuve, Centre Hospitalier Régional Universitaire de Montpellier, Montpellier, France; Queens University Belfast, Ireland

## Abstract

**Objectives:**

Nebulized hypertonic saline (HTS) has beneficial effects including reducing pulmonary exacerbations in Cystic Fibrosis (CF) patients. Several mechanisms may explain these effects but antimicrobial activity of NaCl remains largely unexplored. We aimed to measure the antimicrobial effect of NaCl on *Pseudomonas aeruginosa* isolated from the respiratory tract in CF patients.

**Methods:**

NaCl minimal inhibitory concentration (MIC) and minimal bactericidal concentration (MBC) were determined for strains characterized for mucoidy, antimicrobial resistance, and ability to form biofilm using 0,9% to 15% NaCl solutions. NaCl effects on biofilm formation, preformed biofilm, and mobility were evaluated. Kinetics of antimicrobial effects was studied.

**Results:**

The growth of all isolates (n = 85) from 34 patients was inhibited by 6% NaCl solution. A 10% concentration had a bactericidal activity on 90% of the isolates. Mucoid and multidrug resistant (MDR) isolates displayed lower MICs compared to non-mucoid and to non-MDR isolates, respectively. Time-kill kinetics showed that NaCl exhibited a rapid, dose and growth phase dependent bactericidal effect. Three percent or more of NaCl inhibited biofilm formation for 69% of strongly adherent isolates. A dose-dependent decrease of preformed biofilm viability and an inhibitory activity on bacterial motility were observed.

**Conclusions:**

NaCl inhibited the growth of all isolates and killed 38% of tested isolates within concentration range currently used in therapeutics. Our results suggest that anti-pseudomonal activity is another mechanism of action of HTS to add to those already established. Clinical trials are needed to compare diverse HTS conditions of use (rhythm, dose and mode of delivery) to obtain efficient and optimized anti-*P. aeruginosa* effects. More generally, NaCl effect on other opportunistic pathogens as well as on global microbiotae recovered during polymicrobial diseases warrants further investigations.

## Introduction

Cystic fibrosis (CF) is the most common inherited disease in Caucasians. The disorder is initiated by a defect in the CF transmembrane conductance regulator (CFTR) gene coding for an apical membrane chloride channel mediating transepithelial salt and liquid movement [1]. CF lung disease is characterized by a persistent inflammation and polymicrobial infectious disease evolving throughout life [2]. The chronic persistence of *Pseudomonas aeruginosa* is linked to lung function decline [3]. The consequences of hydro-electrolytic imbalance in the lung have been controversial in the past [1]. Currently, the volume of the airway surface liquid hypothesis suggesting that CFTR defect leads to water loss related to sodium chloride (NaCl) depletion in the mucus remains the main hypothesis [1], [4]. According to this hypothesis, immotile dehydrated plugs are formed leading to inefficient mucociliary clearance that allows bacteria to cause lung infection [4]. This mechanism suggested that hypertonic saline (HTS) nebulization could favor mucociliary clearance [5]. Clinical trials confirmed beneficial effects on lung function and rate of pulmonary exacerbations but no effect on bacterial density was observed [6]–[11]. Testing HTS on a large population of *P. aeruginosa* CF isolates should elucidate the anti-microbial effect of NaCl.

The aim of this study was to characterize the effects of NaCl on 85 *P. aeruginosa* isolates from CF respiratory tract in 34 patients. Results were interpreted according to strains characteristics, i.e., mucoidy, multidrug resistance, and biofilm biomass formation.

## Materials and Methods

### Bacterial strains and collected data

Eighty-five clinical isolates of *P. aeruginosa* have been retrospectively studied (Pa1 to Pa85). Four reference strains, *P. aeruginosa* ATCC 27853, *P. aeruginosa* ATCC 15442, *P. aeruginosa* ATCC 9027, and *P. aeruginosa* CIP A22 were included in the study. Clinical isolates have been recovered over a 6-year period (from 2006 to 2011) from 36 respiratory tract samples in 34 CF patients attending our local CF center (Centre de Ressources et de Compétences de la Mucoviscidose) of Montpellier, France. Patient age ranged from two-month to 61-year old (median, 19 year-old). Chronic colonization was noted for 19 patients with a median period of colonization of 5 years (1 to 14 years). A single isolate was included for 12 patients and multiple colonial morphotypes, i.e., two to six isolates collected either from one or two samples, were tested for 22 patients. Species identification, mucoid status, and antimicrobial susceptibility were retrieved from routine bacteriological sputum analysis. A multidrug-resistant isolate was defined according to the Cystic Fibrosis Consensus Conference of 1994 as an organism resistant to all of the agents in at least of the following groups of antibiotics (ß-lactam agents, amino-glycosides, fluoroquinolones) [12]. HTS nebulization during the study period was noted for each patient.

### Dilution methods used for bacteriostatic and bactericidal activity testing of NaCl

Inoculums for bacteria in stationary and logarithmic growth phases were prepared from MH agar and broth cultures, respectively, by visual comparison to MacFarland standards followed by dilution steps depending on the assay as previously recommended [13]. Minimal Inhibitory Concentration (MIC) determination was performed by 2 different methods, agar (MICa) and microbroth (MICb) dilution methods with bacteria in stationary phase. For MICa, Mueller-Hinton (MH) plates containing 0%, 0.9%, 1%, 2%, 3%, 4%, 5%, 6%, 7%, 8%, 9%, 10%, 12%, and 15% of NaCl were inoculated with a final inoculum of 10^4^ colony forming units (CFU) per spot and incubated one night at 37°C as described previously [13] before reading. For MICb, MH broth containing 0%, 0.9%, 1%, 2%, 3%, 4%, 5%, 6%, 7%, 8%, 9% and 10% of NaCl were inoculated with a final inoculum of 5*10^5^ CFU/ml and incubated one night at 37°C as described previously [13] before reading. Minimal Bactericidal Concentration (MBC) corresponding to the lowest antimicrobial concentration reducing the inoculum by 99.99% within 24 h was determined from MICb assay by subculturing wells, which had no visible growth and the growth control well as described previously [14]. Each inoculum was confirmed by colony count and all assays were performed in duplicate. When different results were obtained in the two independent tests, the higher MIC or MBC value was taken as the result. Time-kill kinetic study of NaCl was performed with a macrobroth dilution method using final inoculum of 5*10^5^ CFU/ml with bacteria in stationary and/or in logarithmic growth phases according to the assay. Inoculation of NaCl-containing broth was performed as described previously [13]. The final test volume was 10 ml of MH and bacterial suspension loads were determined by subculturing serial dilutions of the inoculum after different incubation periods, i.e., 10 min, 30 min, 1 h, 2 h, 3 h, and 24 h, at 4°C or 37°C according to the assay.

### Biofilm formation

The ability to form biofilm biomass was tested according to the crystal violet method [15] and strains were categorized as non-adherent, weakly, moderately, or strongly adherent [15], [16]. The measurement of HTS effect on biofilm biomass formation was performed as described above using a final bacterial inoculum of 5*10^5^ CFU/ml in MH broth containing 0%, 0.9%, 1%, 2%, 3%, 4%, 5%, 6%, 7%, 8%, 9% and 10% of NaCl. All tests were carried out at least three times and results were averaged. The reduction in biofilm biomass formation and hypertonic saline activity on preformed biofilm were evaluated according to the modified method of Pompilio and colleagues [17].

### Evaluation of bacterial motility

Motility of strains in presence of different concentrations of NaCl was globally estimated from diameter of spotted cultures on MH plates used for MICa. A strain was considered as motile when overnight cultures had increased diameter compared to the spot initial size. Motility has been estimated for all isolates for NaCl concentrations lower than MICs obtained by the agar dilution method.

### Statistical analysis

MIC, MBC, and killing quotient (KQ) distributions were analyzed according to the mucoid status, the multidrug resistance, and the ability to produce biofilm. Differences between groups were assessed by Mann-Whitney’s test, with the exception of the evaluation depending on biofilm producer groups, assessed by the Kruskal-Wallis’ test and Spearman correlation coefficient when Kruskal-Wallis’ test was positive. Data were analyzed at the total population level and at a restricted level defined by one isolate type per patient to avoid biased results due to possible clonal relatedness of isolates in a patient because strains have not been genotyped in this study. Statistical analysis of results was done with R project software (http://www.r-project.org). A *P* value ≤0.05 was considered to reflect significance.

### Ethics statements

This in vitro study required neither the agreement of the ethical committee of our institution nor the patient informed consent because it involved only bacterial strains. The study does not involve the sample collection or patient data, and no patient intervention occurred with the obtained results. Only general data patients (age, type of colonization by P. aeruginosa) were reported but no patient-related data were analyzed. No primary human sample materials were used, only bacterial isolates from routine diagnostic procedures or strain collections. All analyzed clinical strains were acquired during routine diagnostic procedures. Accordingly, no acquisition of patient samples for the study was undertaken. We did not conduct research outside of our country of residence.

## Results

### Antibacterial effect of NaCl on the *P. aeruginosa* population

MIC and MBC results are presented in Table 1. Intra and inter-method variability is given in Table S1. For the 85 CF isolates, MIC values ranged from 0.9 to 6%. Depending on the method considered, MIC_50_ and MIC_90_ values were 4 or 5% and 5 or 6%, respectively. The growth of all the isolates was inhibited after a 24 h-incubation with 6% NaCl solution whatever the method considered. Moreover, NaCl displayed a bactericidal effect, 38% of the CF isolates being killed by a 7% NaCl solution, concentration currently used in therapeutics, 50% and 90% of the isolates being killed by 8% and 10% of NaCl, respectively. The effect of NaCl varied depending on the isolate, differences between MICb and MBC value observed for a strain ranging from 1% to up to 7%. No correlation between MICb and MBC values was observed, some isolates displaying low MIC values of 1 or 3% and high MBC values of 7% or 10%, respectively and conversely (data not shown). This resulted in KQ values < 4 for the 83 strains exhibiting MBC values within tested range supporting a bactericidal activity of NaCl on a large majority of strains (Table 1).

**Table 1:** NaCl Minimal Inhibitory Concentration (MIC), Minimal Bactericidal Concentration (MBC) and Killing quotient (KQ) distributions for the 85 CF isolates and the 4 reference strains of *P. aeruginosa.*

	NaCl MIC values (in %)											
	Agar dilution method			Broth dilution method			NaCl MBC values (in %)			Killing quotient (KQ)^a^		
Strains or isolates (n)	MIC_50_	MIC_90_	Range	MIC_50_	MIC_90_	Range	MBC_50_	MBC_90_	Range	KQ_50_	KQ_90_	Range
All strains (n = 89)	4	5	2–6	5	6	0.9–6	8	10	4->10	1.8	2.7	1.2–7.8
Reference strains (n = 4)	5	5	5	5	6	5–6	7	9	7–9	1.4	1.6	1.4–1.6
CF isolates (n = 85)	4	5	2–6	4	6	0.9–6	8	10	4->10	1.8	3	1.2–7.8

MIC_50_ and MIC_90_ values were defined as the lowest concentration of NaCl at which 50% and 90% of the isolates were inhibited, respectively.

a Only isolates exhibiting MBC values within tested range were considered.

### NaCl activity varied according to relevant sub-populations of *P. aeruginosa*

Considering strains recovered in patients with multiple isolates, differences between MICb and/or MBC values ranging from 0 to 3% were observed. Variable response to NaCl with MICb and/or MBC values differing by 3% of NaCl were observed for the isolates recovered from four patients.

Table 2 exhibits MIC, MBC and KQ values of relevant sub-populations. MICb values obtained for mucoid CF isolates were significantly lower than the ones obtained for non-mucoid isolates when the total population (*P*<0.0001) and a subgroup defined by one isolate type, mucoid and/or non-mucoid, per patient (*P* = 0.0003) were considered. MBC distribution did not significantly differ between mucoid and non-mucoid isolate groups when the subpopulation defined by one isolate type per patient was considered (*P* = 0.07) but did when the whole population (n = 85) was considered (*P* = 0.01), which may be related to a lower statistical power in relation to a limited number of strains in the subpopulation (n = 48). KQs for mucoid isolates were consequently higher than those for non-mucoid isolates (*P* = 0.0002 and *P* = 0.0008 for the whole population and the defined subpopulation, respectively) (Table 2). Twenty-two isolates recovered from eleven samples obtained from eleven patients were multidrug resistant (MDR). Considering the whole population (n = 85), non-MDR isolates displayed significantly higher MICb than did the MDR isolates (*P* = 0.0005) while no difference was observed for MBC values. Consequently, KQs were higher for MDR isolates than for non-MDR isolates (*P* = 0.002). A similar trend was observed when considering one randomly selected isolate type, MDR and/or non-MDR, per patient (n = 40) (MICb: *P* = 0.005, KQ: *P* = 0.001) (Table 2).

**Table 2:** Comparison of NaCl Minimal Inhibitory Concentration (MIC), Minimal Bactericidal Concentration (MBC) and Killing quotient (KQ) distributions for subgroups of *P. aeruginosa* CF isolates according to mucoid characteristic, antimicrobial susceptibility pattern, ability to form biofilm and patient under HTS treatment.

			NaCl MIC values (in %)															
			Agar dilution method				Broth dilution method				NaCl MBC values (in %)				Killing quotient (KQ)^a^			
Population	Subgroup	Isolate type (n)	MIC_50_	MIC_90_	Range	*P* value d	MIC_50_	MIC_90_	Range	*P* value d	MBC_50_	MBC_90_	Range	*P* value^d^	KQ_50_	KQ_90_	Range	*P* value d
Total CF isolates	Mucoid characteristic	Mucoid (25)	3	4	2–4	**<0.0001**	3	5	0.9–5	**<0.0001**	7	9	6–>10	**0.01**	2.3	3.5	1.4–7.8	**0.0002**
		Non-mucoid (60)	5	5	2–6		5	6	2–6		8	10	4–>10		1.6	2.3	1.2–4	
	Antimicrobial susceptibility pattern	Multidrug resistance (22)	3	4	2–5	**<0.0001**	3	5	2–5	**0.0005**	8	>10	7–>10	0.3	2.3	3.5	1.4–4	**0.002**
		Non-multidrug resistance (63)	4	5	2–6		5	6	0.9–6		8	9	4–>10		1.8	2.3	1.2–7.8	
	Ability to form biofilm	No (9 of which 3 mucoid)	3	5	2–5	**<0.0001 Rs = 0.44**	3	5	2–5	**<0.0001 Rs = 0.48**	7	9	4–9	0.14	2.3	4	1.4–4	**0.0009 Rs = **–**0.37**
		Weak (42 of which 16 mucoid)	4	5	2–5		4	5	0.9–6		8	10	5–>10		1.8	3.5	1.2–7.8	
		Moderate (18 of which 3 mucoid)	5	5	2–5		5	6	2–6		8	>10	7–>10		1.8	2.3	1.2–3.5	
		Strong (16 of which 3 mucoid)	5	6	3–6		5	6	3–6		8	9	6–10		1.5	2	1.2–2.7	
	Patient under HTS treatment	Yes (5) b	2	3	2–3	ND	3	3	2–3	ND	>10	>10	8–>10	ND	ND	ND	ND	ND
		No (80)	4	5	2–6		5	6	0.9–6		8	9	4–>10		1.8	2.7	1.2–7.8	
One isolate per type, per patient c	Mucoid characteristic	Mucoid (18)	3	4	2–4	**<0.0001**	3	5	2–5	**0.0003**	7	9	7–>10	0.07	2.3	3.5	1.4–4.5	**0.0008**
		Non-mucoid (30)	5	5	2–6		5	6	2–6		8	9	5–>10		1.5	2.3	1.2–3.3	
	Antimicrobial susceptibility pattern	Multidrug resistance (11)	3	4	2–5	**0.002**	3	5	2–5	**0.005**	8	10	7–>10	0.6	2	3.5	1.6–3.5	**0.001**
		Non-multidrugresistance (29)	5	5	2–6		5	6	0.9–6		8	9	5–>10		1.5	2.3	1.2–7.8	
	Ability to form biofilm	No (7 of which 1 mucoid)	3	5	2–5	**<0.0001 Rs = 0.5**	3	5	2–5	**0.0004 Rs = 0.46**	7	9	4–9	0.12	2.3	4	1.4–4	0.056
		Weak (24 of which 8 mucoid)	4	5	2–5		4	6	2–6		8	10	5–>10		1.6	2·5	1.3–3.5	
		Moderate (15 of which 3 mucoid)	5	5	2–5		5	6	2–6		8	>10	7–>10		1.8	2·3	1.3–3.5	
		Strong (11 of which 2 mucoid)	5	6	4–6		5	6	4–6		9	9	7–10		1.5	2	1.3–2	
	Patient under HTS treatment	Yes (1)	ND	ND	2	ND	ND	ND	3	ND	ND	ND	>10	ND	ND	ND	ND	ND
		No (33)	5	5	2–6		5	6	2–6		8	9	5– >10		1.6	2.5	1.2–3.5	

MIC_50_ and MIC_90_ values were defined as the lowest concentration of NaCl at which 50% and 90% of the isolates were inhibited, respectively.

a Only isolates exhibiting MBC values within tested range were considered.

b 5 isolates recovered in one patient.

c isolates were randomly selected.

d Comparison of MIC, MBC and KQ distribution between subgroups was performed with Mann-Whitney’s test, with the exception of the evaluation depending on biofilm producer groups, assessed by the Kruskal-Wallis’ test, and Spearman correlation coefficient (Rs) when Kruskal-Wallis’ test was positive. A *P* value ≤0.05 was considered to reflect significance and was indicated in bold type.

ND, not determined either because only one isolate or one patient was included in the sub-population, or because one isolate exhibiting a MBC value within the tested range.

Five strains were recovered (Pa17 to Pa21) from one patient under 6% HTS treatment administered once or twice daily for 4 years. These isolates displayed low MICb values (3%, 3%, 2%, 3%, and 3%, respectively) and high MBC values (>10%, >10%, 8%, >10%, and >10%, respectively).

### NaCl mechanism of action

#### Time-kill kinetics

**Figure 1:**
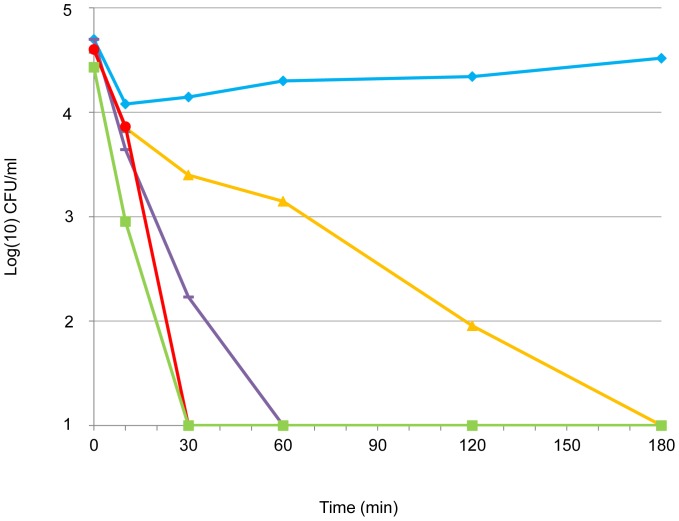
Time-kill kinetics of different NaCl concentrations against *P. aeruginosa* isolate Pa71. 3% NaCl solution (orange line, filled triangles), 5% NaCl solution - corresponding to the MBC value (violet line, dash), 7% NaCl solution (red line, filled circles), and 10% NaCl solution (green line, filled squares). Control (blue line, filled lozenges) was not exposed to NaCl. Time-kill curves of 15% (not shown) and 10% NaCl solution were similar.

**Figure 2:**
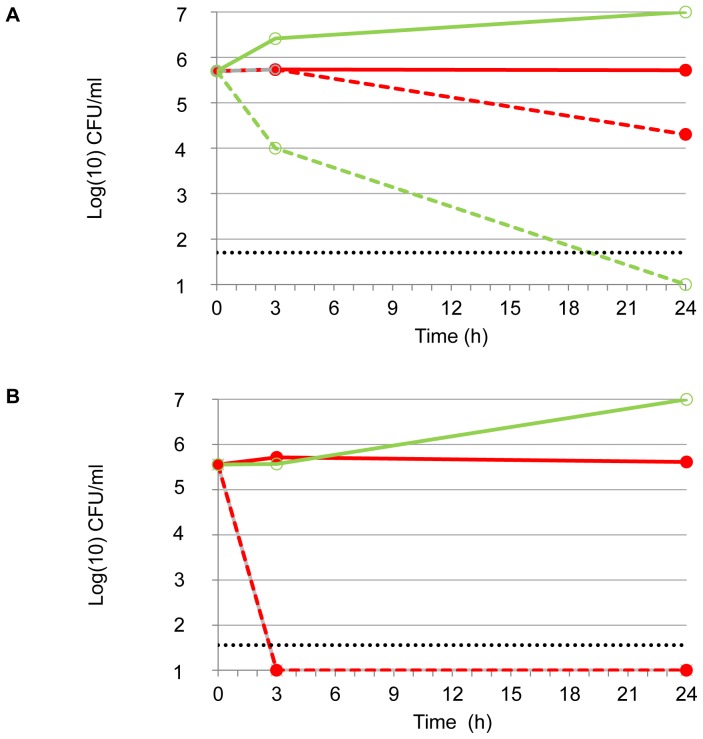
Time-kill kinetics of NaCl against *P.aeruginosa* Pa1 according to bacterial phase of growth and incubation temperature. Inoculum in stationary phase of growth (A) and in logarithmic phase of growth (B) for Pa1 (NaCl MBC: 7%). Bacterial suspension in NaCl at the MBC value were incubated at 37°C (green dotted line, open circles) or 4°C (red dotted line, filled circles). Controls (full lines) were not exposed to NaCl and were incubated at 37°C (green line, open circles) or 4°C (red line, filled circles). The horizontal dotted line indicates a 4-log reduction in viability. B: dotted green (open circles) and red (filled circles) lines are superposed.

Time-kill kinetics was performed on two strains selected for divergent phenotype, i.e., strain Pa1 (mucoid, MICb of 3%, MBC of 7%) and strain Pa71 (non-mucoid, MICb of 2%, MBC of 5%). Inoculum in logarithmic phase of growth was tested for isolate Pa71 against various concentrations of NaCl (Figure 1). Time-kill curves highlighted that a rapid decrease in inoculum size was observed from 30 min and before 3 h of contact with NaCl. NaCl antibacterial activity under *in vitro* conditions was shown to be both concentration- and time-dependent. Impact of both growth phase and temperature on time-kill kinetics of NaCl was evaluated on isolate Pa1 (Figure 2). The Pa1-time-kill curve also showed a rapid NaCl effect when bacteria in logarithmic phase of growth were incubated at 37°C in presence of NaCl at the MBC. With bacteria in stationary phase of growth tested in the same conditions, the killing effect was also observed but was drastically delayed (Figure 2) and the bactericidal effect of 4-log reduction of the inoculum was absent within 24h when the assay was performed at 4°C (Figure 2). Altogether, these results suggested that anti-*P. aeruginosa* activity of NaCl might be different than a mere osmotic effect because it depended on temperature conditions and physiological bacterial state.

#### Effects of NaCl on biofilm

All reference strains were able to form biofilm biomass, being either strongly, moderately, or weakly adherent. The majority of the 85 CF *P. aeruginosa* isolates were able to form biofilm biomass (n = 76) but most of these isolates were weakly adherent (n = 42) (Table 2). Most mucoid isolates were weakly adherent (Table 2). Among the 22 patients colonized by two or more *P. aeruginosa* colony types, five harbored isolates showing a unique adherence type and 13 had isolates showing two adherence types. More rarely, isolates recovered from a same patient may present three or four adherence types (two patients each).

We found that MICb values were correlated to the ability to form biofilm biomass, the strongly adherent isolates being associated with higher MICb values when both the whole population and a subgroup defined by one randomly selected isolate per type per patient were considered (*P*<0.0001 and *P* = 0.0004, respectively) (Table 2). There was no significant trend between the MBC value and the level of adherence (*P* = 0.14 and 0.12 respectively) (Table 2).

**Figure 3:**
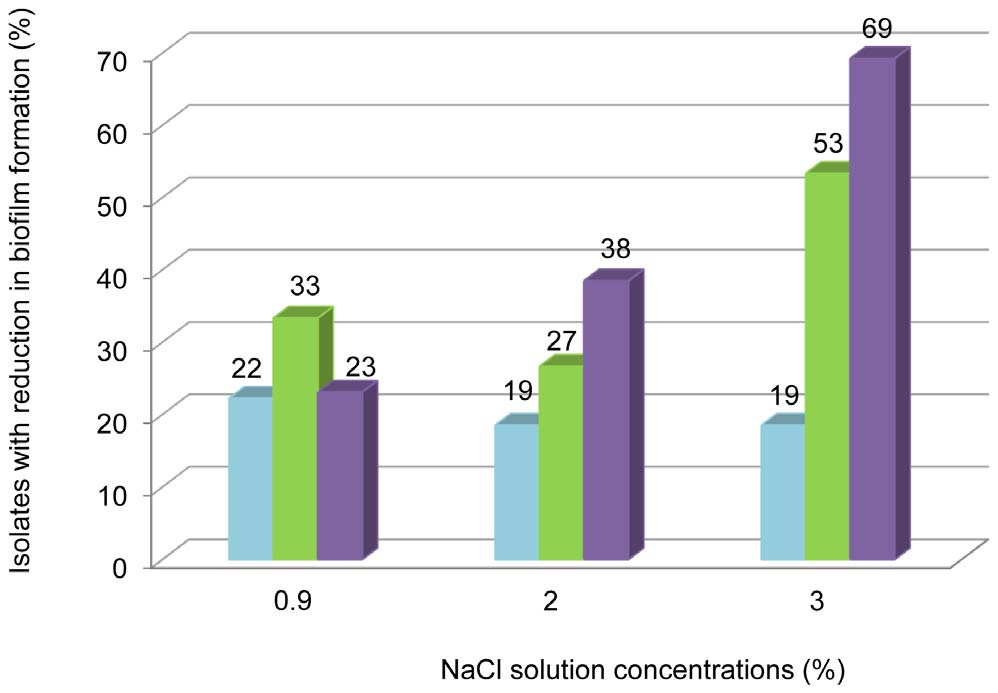
Effect of three sub-inhibitory concentrations of NaCl against *P. aeruginosa* CF isolate biofilm biomass formation. Tested for 55 adherent CF isolates of *P. aeruginosa* with MIC values > 3% distributed as follows: strongly adherent (n = 13, violet bars), moderately adherent (n = 15, green bars), weakly adherent phenotype (n = 27, blue bars). Prevention of biofilm biomass formation is presented as percentage of strains for which ability to form biofilm biomass was decreased by at least 25% compared to controls (not exposed).

A reduction of at least 25% of biofilm biomass formation was highlighted for 23% to 69% of strongly adherent strains depending on the NaCl concentration with a dose-dependent effect, and for 27% to 53% of moderately adherent isolates (Figure 3). Twenty percent of weakly adherent isolates showed a decrease in their ability to form biofilm biomass independently of the NaCl concentration tested. With 2 and 3% of NaCl, rates of isolates showing reduction in biofilm biomass formation increased with the adherence level. Analysis conducted on subgroups defined by one isolate per type of adherence per patient showed similar results (data not shown).

**Figure 4:**
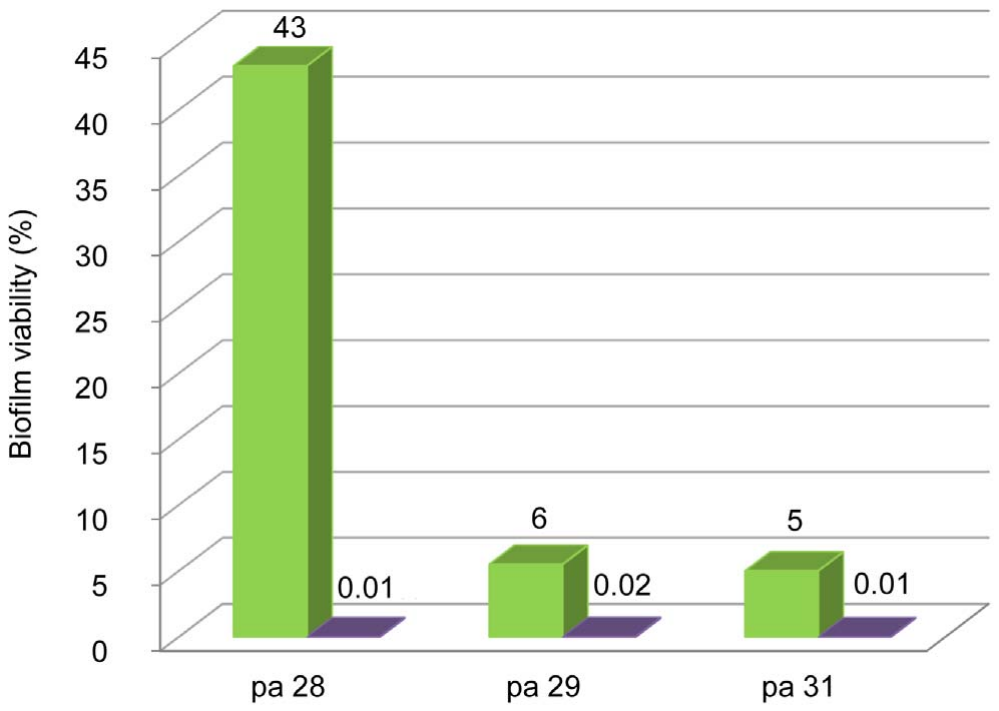
Activity of 7% and 10% NaCl solution against preformed biofilm for three strongly adherent isolates. 7% (green bars) and 10% (violet bars) NaCl solution was tested against preformed biofilm for the three *P. aeruginosa* CF isolates Pa28, Pa29, and Pa31. Results are expressed as percentage of biofilm viability compared to non-exposed control after 24 h of NaCl exposure.

*In vitro* activity of NaCl solution (7% and 10%) was evaluated on preformed biofilm for Pa28, Pa29, and Pa31, three strongly adherent, non-mucoid, non-MDR isolates with identical MICb and MBC values (MICb = 6%, MBC = 9%) recovered from three patients. Biofilm viability was reduced and this appeared to be dose dependent (Figure 4).

**Figure 5:**
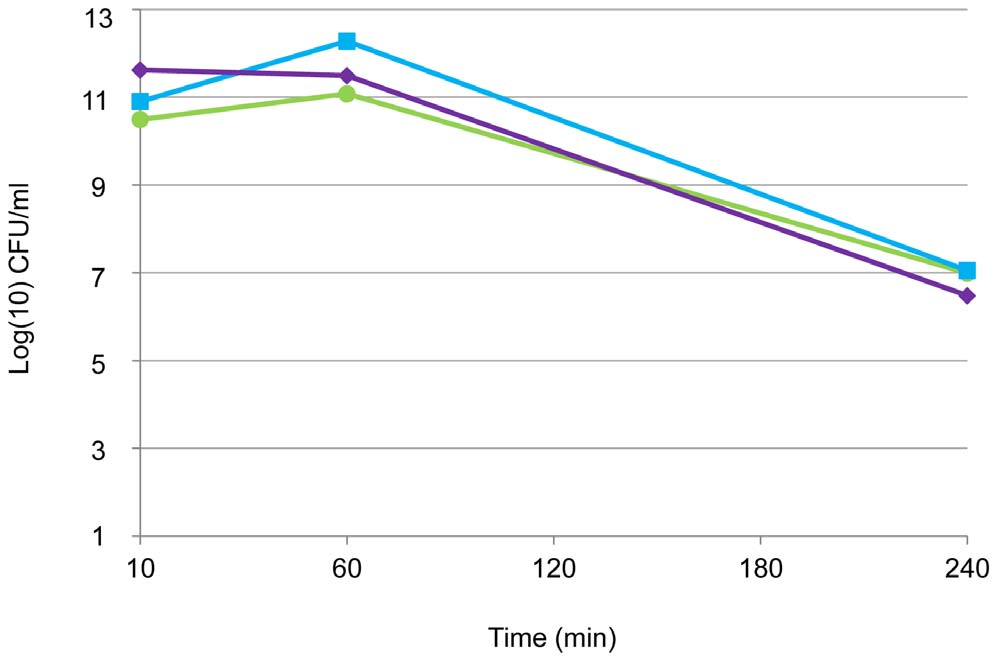
Time-kill kinetics of 10% NaCl solution against preformed biofilm for three strongly adherent CF isolates. *P. aeruginosa* CF isolates: Pa28 (green line, filled circles), Pa29 (blue line, filled squares), Pa31 (violet line, filled lozenges).

Time-kill kinetics showed a killing effect on preformed biofilm resulting in at least a 10^−3^ decrease in bacterial load and occurring between 1 h and 4 h of contact with 10% of NaCl (Figure 5).

#### Evaluation of NaCl activity on motility

**Figure 6:**
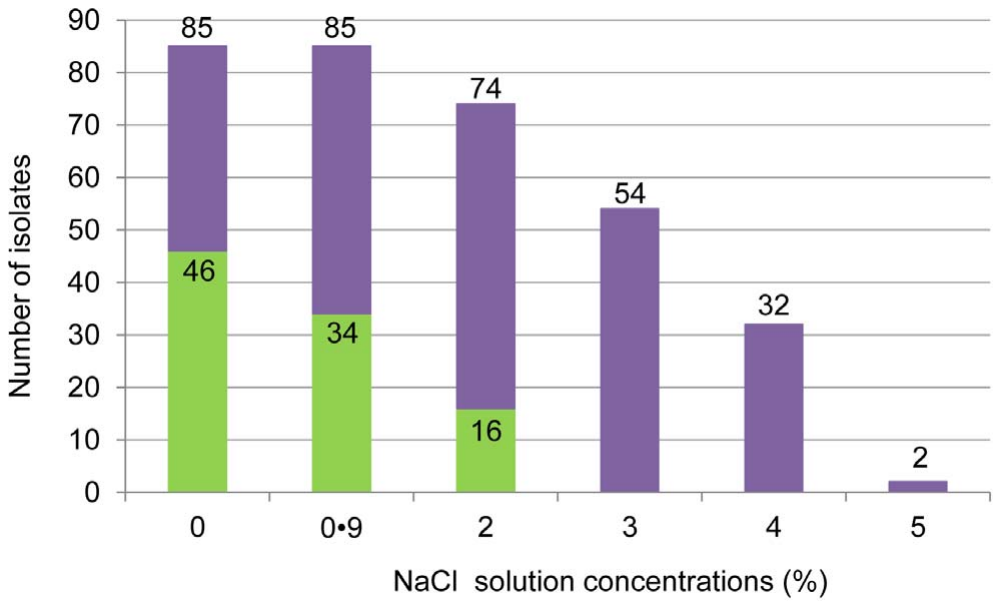
Rate of motile isolates according to NaCl concentration. Green bars show the number of motile isolates among isolates with non-inhibited growth (full bars).

For NaCl concentration above 2%, none of the 46 isolates displaying motility in the absence of NaCl were motile anymore (Figure 6), suggesting that NaCl inhibited isolate motility at concentrations lower than those inhibiting bacterial growth.

## Discussion

Short-term and long-term clinical randomized trials in CF patients showed beneficial effects of HTS nebulization on lung function in reducing exacerbations or on the perception of effectiveness of chest physiotherapy [6], [8], [9], [11]. Positive effects were explained by mucus rehydration and mucociliary clearance improvement, anti-inflammatory activity and indirect effects by activation of antimicrobial peptides [4], [5], [18]. No report investigated antimicrobial activity of NaCl as a possible explanation supporting beneficial effects of such a treatment.

We proposed the first study evaluating *in vitro* NaCl activity on a large population of *P. aeruginosa* isolated from CF patients. Multiple antimicrobial effects of NaCl on CF *P. aeruginosa* are demonstrated with NaCl concentrations used in therapeutics. Indeed, we showed that: i) 6% NaCl solution inhibits bacterial growth for all the tested isolates, ii) a killing effect was observed from 4% NaCl solution, and iii)10% NaCl solution had a bactericidal activity on 90% of the isolates. We also demonstrated that NaCl had effects on bacterial biofilm being able to alter biofilm biomass formation of strongly adherent isolates at sub-inhibitory concentrations and to have a killing effect on preformed biofilm. However, according to MIC values, isolates with strong biofilm biomass production abilities were more resistant to NaCl action suggesting that the mature biofilm acts as a protection against inhibitory effect of NaCl. Finally, although the method used in this study did not allow study of the type of motility, i.e., swarming, swimming or twitching motility, affected by NaCl, we showed that NaCl inhibited the strain global motility as previously suggested for four *P. aeruginosa* strains [19]. Since bacterial motility and loss of motility are known to play a role in biofilm formation by *P. aeruginosa* [20], the specific impact of the motility inhibition by NaCl on biofilm formation has still to be evaluated. Time kill kinetics evaluated *in vitro* for 2 selected isolates suggested that NaCl antibacterial activity was both concentration- and time-dependent. Of note, these results were observed even though using the most stringent definition of the MBC, i.e., the lowest antimicrobial concentration reducing the inoculum by 99.99% within 24 h and bacterial growth conditions, i.e., bacteria in stationary phase of growth, which could have led to overestimation of MBC for some isolates. Of particular interest is the higher susceptibility to NaCl observed for clinically relevant subpopulations of *P. aeruginosa*, i.e., mucoid and/or multidrug resistant isolates. We found that the growth of mucoid isolates was inhibited by significantly lower NaCl concentrations than the one of non-mucoid isolates, as described for a *mucA22* mutant that was significantly more sensitive to osmotic stress than the isogenic parental *P. aeruginosa* PAO1 strain [21]. These results suggested that the reduction in exacerbation rate following prolonged treatment with HTS nebulization resulted from a population shift from mucoid to non-mucoid *P. aeruginosa* [21], [22]. MICs were also significantly lower for MDR isolates than non-MDR isolates. Although not further investigated here, this could be related to a fitness cost of multidrug resistance [23] that could impair osmotic stress response. Altogether, active antimicrobial activity of NaCl against CF *P. aeruginosa* isolates might contribute to the *in vivo* global positive effect of HTS treatment. In addition, indirect effects may also enhance NaCl antimicrobial effect *in vivo* like disruption of inhibitory interactions between glycosaminoglycans present within the mucus and antimicrobial peptides [24].

HTS treatment is currently not standardized and varies according to the study in the volume of aerosolized HTS (4, 5, or 10 ml), the rhythm of administration (twice or four times daily), the mode of administration (via jet or positive expiratory pressure nebulizers), and the HTS concentration (5.85%, 6%, or 7%) [6], [8], [9], [11], [25]. The effect of HTS appeared to be both dose-dependent [26] and time-dependent [27]. Considering the absence of significant change in the density of *P. aeruginosa* observed in a previous study [6], it could be assumed that the conditions of treatment, i.e., 4 ml of 7% HTS twice daily, were not bactericidal but only inhibitory against *P. aeruginosa in vivo.* Our results open important areas of research by suggesting that optimization of HTS conditions of use (rhythm, dose, mode of delivery) in CF may contribute to enhance anti-*P.aeruginosa* effects and thereby treatment clinical efficacy. However, studies that use *in vivo* models and randomized clinical trials are needed to evaluate the performances of diverse HTS administration conditions and to determine the more appropriate and efficient conditions of HTS use in CF patients. More largely our study highlights the potential use of NaCl as a basic but important component of the therapeutic arsenal used in the management of infectious diseases. Hyperosmotic stress is known for reducing biofilm growth and for being bactericidal on *Escherichia coli*, *P. aeruginosa,* and *Enterococcus faecalis* when NaCl is used at 13% to 35% and could be used for treatment of root canal infection [28] or for locking tunneled hemodialysis catheters [29]. Considering the lower NaCl concentrations used in this study, the antimicrobial effect was probably not related to a direct hyperosmotic lysis. We presented arguments for an antimicrobial activity linked to an active biological process: i) NaCl was efficient mainly on bacteria in logarithmic phase of growth independently of the temperature and to a lesser extent on bacteria in stationary phase of growth at 37°C, ii) in stationary phase of growth at 4°C, which could be considered as metabolic standby conditions, HTS produced slight antimicrobial effects on *P. aeruginosa*, iii) variable response to HTS was observed among the population, and iv) loss of motility occurred from 2% NaCl solution. In summary, because the response to NaCl depended on the bacterial physiological status and varied among the population of *P. aeruginosa*, we assumed that NaCl bactericidal effect could be due to complex regulated mechanisms rather than to a mere hyperosmotic effect.

Several bioclinical answers remain to be addressed. Particularly, longitudinal studies are needed to monitor the evolution of NaCl MICs and MBCs of *P. aeruginosa* isolates recovered from the respiratory tract in patients under HTS treatment. In this study, only one patient was under HTS treatment for 4 years and isolates displayed high MBC and low MIC values. For this purpose, methods first proposed in this study for NaCl antimicrobial activity evaluation appeared suitable because of their good reproducibility. In addition, evaluating the NaCl action on several other CF pathogens including viruses, yeast, and fungi would be of interest because recent studies suggested NaCl-altered growth beyond NaCl concentration used in therapeutics for some viruses and yeasts not involved in CF [30], [31]. The effect of HTS on polymicrobial community of the CF respiratory tract should be investigated not only because halotolerant bacteria may be favored by such a treatment but also because any modification of the relative composition of this complex microbiota will impact intercellular interactions and communication involved in this polymicrobial disease with unknown consequences [2], [32]. Finally, the antimicrobial mechanism of action of NaCl warrants further investigation because it is still not fully elucidated, particularly the hypothesis of the possible induction of lytic activity of *P. aeruginosa* bacteriophages by NaCl has to be explored. Indeed, several environmental factors such as temperature or pH but also salinity could influence the transition from lysogenic to lytic phage [33], [34].

Over the last decade, treatment strategies have shifted from controlling chronic infection to attempting to eradicate *P. aeruginosa* in the early stages of infection. Whatever the strategy, the antibiotic resistance makes the need for alternative approaches obvious and non-antibiotic treatments may expand the therapeutic repertoire. NaCl, as a simple and basic compound, may become a pivotal resource in CF care and in fighting *P. aeruginosa*. Beside polyvalent effects on the CF respiratory tract, we gave insights into antimicrobial activities of NaCl. NaCl kills *P. aeruginosa* but also acts on a range of its core eco-physiological functions such as biofilm formation and motility. Future studies should go further toward evaluating the role of NaCl in bacteriophage predation. Taken together, results and hypotheses on NaCl effects open research paths to treat CF as an eco-pathology of the respiratory tract. More widely, the efficacy of NaCl alone or in association with current available therapeutics should be more deeply investigated in infectious diseases.

## Supporting Information

Table S1 **Reproducibility and inter-method variability of Minimal Inhibitory Concentration (MIC) and Minimal Bactericidal Concentration (MBC) measurements.**
(DOC)
