# Supplementary material for: Advances toward the Elucidation of Hypertonic Saline Effects on Pseudomonas aeruginosa from Cystic Fibrosis Patients
Source: PLoS One. 2014 Feb 28;9(2):e90164. doi: 10.1371/journal.pone.0090164 (PMC3938589; doi:10.1371/journal.pone.0090164)
Supplement: Table S1 — Reproducibility and inter-method variability of Minimal Inhibitory Concentration (MIC) and Minimal Bactericidal Concentration (MBC) measurements. (DOC) [file pone.0090164.s001.doc]

**Table S1: Reproducibility and inter-method variability of Minimal Inhibitory Concentration (MIC) and Minimal Bactericidal Concentration (MBC) measurements.**

___________________________________________________________________________

**Assay Method Difference between MIC or Strain**

(strain No.) (strain No.) **MBC values (% of NaCl) No. %**

___________________________________________________________________________

**MIC determination Agar dilution** 0 64 71.9

(89) (89) 1 21 23.6

2 4 4.5

_________________________________________________________

**Microbroth dilution** 0 51 57.3

(89) 1 32 36

2 6 6.7

_________________________________________________________

**Inter-method variability** 0 45 50.5

(89) 1 40 45

2 4 4.5

___________________________________________________________________________**MBC determination** 0 45 54.2

(83^a^) 1 32 38.6

2 6 7.2

___________________________________________________________________________

^a^ Six isolated were excluded on the basis of at least one MBC measurement >10%.
